# Comparing reflection levels between facilitator-led and student-led debriefing in simulation training for paramedic students

**DOI:** 10.1186/s41077-023-00273-0

**Published:** 2023-12-14

**Authors:** Carl Robert Christiansen, Jeanette Viggen Andersen, Peter Dieckmann

**Affiliations:** 1 Department for Prehospital Education and Research, Faculty of Health Sciences, Oslo Metropolitan University, Oslo, Norway; 2 Department of Quality and Health Technology, Faculty of Health Sciences, University of Stavanger, Stavanger, Norway; 3 Copenhagen Academy for Medical Education and Simulation (CAMES), Center for Human Resources and Education, Herlev and Gentofte Hospital, Herlev, Denmark; 4 Department of Public Health, Copenhagen University, Copenhagen, Denmark

**Keywords:** Debriefing, Education, Healthcare Simulation, Paramedic Student, Peer-Assisted Learning (PAL), Peer-Assisted Simulation (PAS), Reflection

## Abstract

**Background:**

Simulation in healthcare attempts to create relevant representations of patient encounters. It provides experiential learning, bridging typical classroom activities and clinical practice. This study aims to investigate whether the principle of Peer-Assisted Learning can be used in simulation by letting simulation-experienced paramedic students prepare, deliver, and debrief their own simulations, with minimal faculty assistance. This could be a way to support student learning by being involved in teaching, and it might at the same time optimise the cost-effectiveness of simulation-based training.

**Methods:**

This observational non-inferiority study compared reflection levels between facilitator-led and student-led simulation and debriefing, between scenario types, and compared the number of turns in which students are involved in both settings. Third-year Bachelor in Paramedic Science students’ debriefings were filmed and transcribed. The degree of reflection in students’ statements was rated according to a modified version of Fleck’s analytical framework of reflection levels, assigning scores from lowest (R0 description) to highest (R4 critical reflection). Facilitator-led and student-led debriefings were compared using chi-square tests. Scenarios were also analysed according to type (paediatric emergencies and complex assessments) regardless of who led the simulation.

**Results:**

Ten facilitator-led and 12 student-led debriefings were analysed. Students gave 682 (49%) contributions in the facilitator-led debriefings, and 702 (51%) contributions in student-led debriefings. Comparison of reflection levels between facilitator-led and student-led debriefings was respectively: R0-level 32.7% vs 33.8%, R1-level 44.0% vs 44.3%, R2-level 14.7% vs 17.1%, R3-level 0.1% vs 1.3%, and R4-level 0.1% vs 0.1%. There were no statistically significant differences in reflection levels between facilitator-led and student-led debriefings (*p* = 0.178). Comparing the reflection levels between the scenarios on “paediatric emergencies” and “complex assessments”, the results were respectively: R0-level 35.4% vs. 31.7%-level, R1-level 45.3% vs. 43.3%-level, R2-level 13.4% vs. 17.8%, R3-level 0.5% vs. 0.9%, and R4-level 0.0% vs. 0.3%. These differences were statistically significant (*p* = 0.010). No significant differences in engagement were found between debriefings led by a student or a facilitator, when measuring the number of turns in the conversations.

**Conclusions:**

Facilitator-led and student-led debriefings resulted in equivalent reflection levels amongst students. Student-led simulation is potentially a cost-effective supplement to regular simulation within a healthcare degree program. Since complex scenarios provided higher reflection levels than paediatric, scenario design might influence reflection levels.

**Supplementary Information:**

The online version contains supplementary material available at 10.1186/s41077-023-00273-0.

## Introduction

Simulation in healthcare is a learning strategy that attempts to create a relevant, goal-oriented representation of a patient encounter, which allows learners to train a clinical situation and reflect thereupon. Role-playing, simulation tools (e.g. mannequins, special monitors), medical equipment and a mock environment may be used to achieve this [1]. Simulation allows educators to control the clinical situation and learning environment, according to participants’ learning needs and curricular requirements. Objectives can be to train practical procedures, decision making, teamwork and other topics in a safe and reproducible manner [2]. The intent is to provide opportunities for learning that can be applied to patient care, creating a link between typical classroom activities and clinical practice [1, 3]. A key actor in this learning setting is the facilitator. This is a simulation-trained professional who enables the simulation itself and guides the participants through the post-simulation reflective process known as debriefing [4]. The debriefing is an essential element of experiential learning and can be defined as a “discussion between 2 or more individuals in which aspects of a performance are explored and analysed with the aim of gaining insights that impact the quality of future clinical practice” [5]. It is a structured conversation where the experiences are put into perspective and linked to prior knowledge. Experiential learning would be random if it was not for a debriefing [6, 7]. Simulation is a costly endeavour because the facilitator-student ratio is high, in addition to expenses of equipment, wear and tear, medical consumables and facilities [8, 9].

The idea of student-led simulation was founded on the principle of Peer Assisted Learning (PAL) where students learn from other students [10]. Previous studies have shown students to be effective teachers [11]. PAL involves members of comparable social groups who are not trained teachers helping one another learn by teaching each other. This could be colleagues, students at different academic year levels, or students within the same academic year level. There are many variations of PAL, which can be classified according to group sizes (one-to-one, one-to-few or one-to-many), and the relationship between the learners (peer-to-peer or peer-to-near peer) [10, 12, 13]. PAL is believed to be qualitatively different from teacher-led learning, with different benefits and drawbacks. The benefits include increased comprehension and knowledge retention; improved non-technical skills and communication abilities; and improved self-direction and learning processes. Potential drawbacks can be insufficient time to prepare; uncertainties regarding the extent of curriculum content covered; issues with group dynamics; varying learning paces amongst students; student anxiety; and the pooling of knowledge gaps when students of insufficient understanding teach each other [12–14].

In studies addressing facilitator and non-facilitator-led debriefing, some report superior effect when debriefing is led by a facilitator [15, 16], whilst others report no meaningful difference [17–19]. However, studies are difficult to assess as nomenclature is heterogeneous and lacks clarity. For example are self-debriefing [16, 17], unfacilitated debriefing [15] and peer-led debriefing [16] used to describe non-facilitator-led debriefing. These terms do not distinguish if a dedicated peer is appointed to lead the debriefing whether debriefing is an open group process, and further if this peer has an observatory or participatory role in the scenario. Regardless of the ambiguity, some risks have been identified with non-facilitator-led debriefing, here defined as any debriefing approach not led by a trained facilitator. These are risks of the debriefing primarily containing commonly known information and missing points which are only known by a few individuals [20, 21], and that a minority of team members might dominate the dialogue [15]. Benefits on the other hand are increased student engagement; promotion of leadership, communication skills, and confidence [22]; letting participants pace discussions according to needs; and to a larger extent letting students address self-perceived issues [23].

Several studies have investigated PAL in simulation within healthcare education. This article refers to the concept as *student-led simulation*. Studies report different practical approaches. Some let participants script their own scenarios [24, 25], some have faculty involvement and quality assurance in the scripting process [26], whilst others provide students with faculty-scripted scenarios [27, 28]. Another variation is students alternating between delivering simulations and debriefings to each other [24, 26, 27], and student groups doing simulation alone and then self-debrief [28]. There are also differences in same-cohort [26, 27] or mixed-cohort student groups [24, 28]. Common for all identified studies is that participating students were towards the end of their education, and the use of a medium fidelity approach to simulation [24–28]. In this setting, fidelity refers to the degree of functional realism the simulation mannequins and other simulation equipment can achieve [29]. Outcome measures were mostly student self-reporting with Likert scale questionnaires. In all these studies students either agreed or strongly agreed that PAL in simulation improved learning, was a positive learning experience, and increased self-confidence [24, 26–28]. One study reported that writing scenarios was educationally valuable [24]. A limitation is that no study reported to what extent this translated to actual learning, behavioural changes, or improved clinical outcomes. So far, no study has demonstrated an association between students positive self-reporting on the reaction level with higher-level outcome measures like learning, behavioural change or clinical outcome [30]. These studies do however show positive indication of using PAL in simulation, and this warrants further investigation into this approach.

Reflection is to look backward at a past event and analyse it with the intent of learning to improve future practice [31–34]. Donald Schön linked the ability to reflect on experiences to professional competence. In his view, scientific knowledge’s technical rationality is alone insufficient to meet the indeterminate reality of professional practice. Professionals also need the artistry to apply knowledge to the practical real world, and this artistry is developed through reflecting on experience [35]. Hence, the ability to reflect on practice might be a useful indicator of professional competence. Debriefing relates to reflection as it is the arena where participants do their reflective work. In a sensemaking process, the simulation experience is recontextualised through storytelling, perspective sharing, evaluation, and discussion. This leads to a new understanding of future roles and clinical understanding [36]. Although it is the participants who do the reflective work, one study demonstrated that debriefers can trigger this through certain questions [37], while another study was not able to make a strong link between the reflections shown and questions asked [38].

Debriefing is a complex social practice and many different angles could be used to investigate it [39, 40], including for example, psychological safety [41, 42], interaction patterns [43, 44], learning processes [45], or of the roles involved [46].

Besides the pedagogical benefits, student-led simulations are potentially more cost-effective, as a lower number of faculty is required to produce a simulation of sufficient quality. While it would be problematic to implement such an approach to save money, reducing the cost of simulation-based training would allow it to be more widespread. If the learning outcome would be still good enough, this might be a win-win situation.

Because of the aforementioned potential benefits of student-led simulation-based learning, this study aimed to narrowly investigate whether students could prepare and deliver healthcare simulation, with learning outcomes grounded in a university degree program. This was investigated by evaluating student’s level of reflection as demonstrated by their contributions in simulation debriefings. The hypothesis was that student-led simulation would achieve equivalent reflection levels in debriefings when compared to facilitator-led simulation. This was based on our own experiences with student-led simulations, where we observed students discuss with each other and where we frequently saw deep-going reflections. Also, as we unfolded above, literature on PAL and research on learning in peer groups support this assumption [19, 47–50]. A secondary aim was to investigate whether the type of scenario affected students’ reflection levels in debriefings. The hypothesis was that students would achieve equivalent levels of reflection in debriefings, regardless of simulation scenario type. Additionally, we wished to investigate the extent to which students’ contributions in the debriefing conversation were affected depending on whether they were led by a facilitator or a peer student.

## Methods

This is an observational non-inferiority study [51] comparing reflection levels between facilitator-led and student-led simulation and debriefing, between scenario types, and comparing the number of turns in which students are involved in both conditions.

### Study context

The study was performed at Oslo Metropolitan University (OsloMet) in conjunction with regular simulation activities at the bachelor program in Paramedic Science. The program utilises simulation extensively with groups of 5–7 students simulating a scenario, and for this, every group requires one facilitator. The faculty wished to increase simulation activity but resource constraints required experimentation with alternative approaches. As facility and equipment incur fixed costs, while staff is considered a variable cost, options for reducing staff presence were investigated. This led to the novel concept of student-led simulation where students wrote their own scenario scripts, and then facilitated the simulation and debriefing for some of their fellow students, thus, reducing the need for staff. Today student-led simulation is routinely arranged towards the end of the 3rd, 4th, and 5th semesters. Simulation is an obligatory learning activity, consequently, students have to participate in order to pass the course. Since students have to both provide and participate in simulation, they may also be motivated by reciprocity towards their fellow students to arrange valuable simulation experiences. Students are given selected topics from the curriculum to expose them to key concepts throughout the simulation day when rotating between scenarios.

**Fig. 1:**
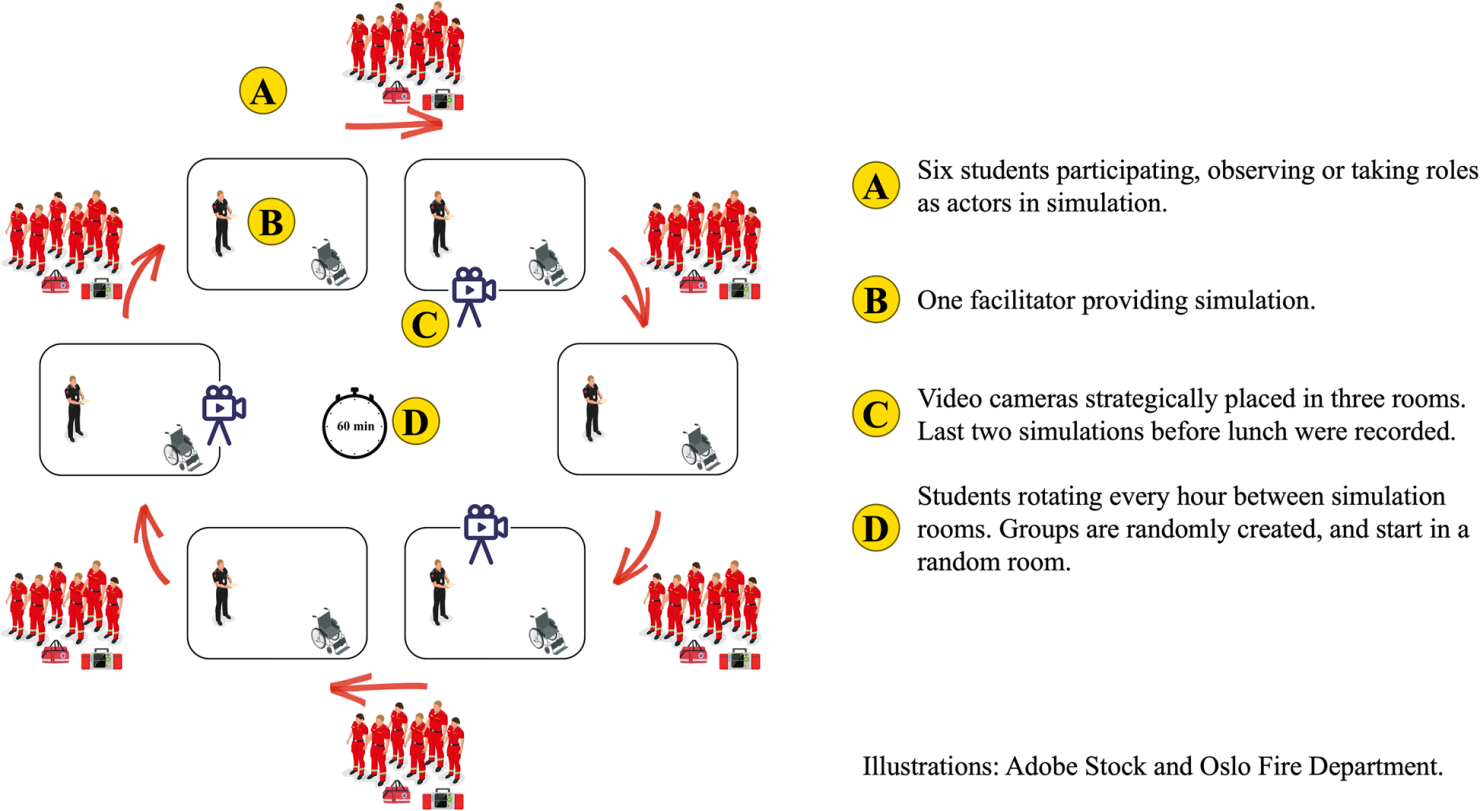
Organisation of facilitator-led simulation days

The study population was 45 third-year paramedic students (23 female, and 22 male; median age 23 years, range 21 to 34 years), and the team of 25 clinically active paramedic facilitators who hold part-time employment at the program. Facilitators had all previously completed a 3-day course on how to facilitate simulations and had between 1- and 4-year experience as facilitators. For debriefing, facilitators are taught to use the Steinwachs model which consists of setting the scene, a descriptive phase, an analytical phase, and an application phase [52]. A group of faculty members standardises and curates the scenarios that facilitators provide, including a debriefing guide. Of the 170 scenarios delivered by facilitators in the study period, ten were conveniently sampled and their debriefings filmed. This comprised eight different facilitators and four different scenario scripts. As we wanted to compare types of scenarios, we selected two scenarios from the paediatric simulation day (1 and 2 in Table 1), and two from the complex assessments´ simulation day (3 and 4 in Table 1). The paediatric scenarios were about life-threatening medical conditions. The complex assessment scenarios were about vulnerable and/or multimorbid patients with compound issues of a medical, legal, ethical, social, and/or practical nature. See Fig. 1 for organisation of facilitator-led simulation.

**Table 1:** Scenario overview with topic and learning focus

Facilitator-led	
1	*Child with septic shock* Assessment, decision making and management of child with septic shock.
2	*Child with hypoglycaemia* Assessment, decision making and management of the child with hypoglycaemia.
3	*Nursing home resident with complex needs* Scenario with frail, multi-morbid nursing home patient with uncertain end-of-life situation, and concomitant hypoglycaemia.
4	*Frail geriatric patient refusing help after fall* Scenario with a frail and alcoholic patient with frequent falls. Uncertainty surrounding coping of activities of daily life and mental capacity.
Student-led	
5	*Reduced level of consciousness in children* Scenario related to a child with a reduced level of consciousness. The focus should include assessment, management, and communication with parents.
6	*Difficulty of breathing in children* Scenario related to a child with difficulty of breathing. The focus should include assessment, management, and communication with parents.
7	*Vulnerable patient group* Scenario related to a vulnerable patient group. The focus could include practical, communicative, medical and/or ethical dilemmas.
8	*Geriatric patient with complex needs* Focus could include frailty, polypharmacy, ethical dilemmas, cooperation with other health care professionals and/or triage to health- and social care service.

**Fig. 2:**
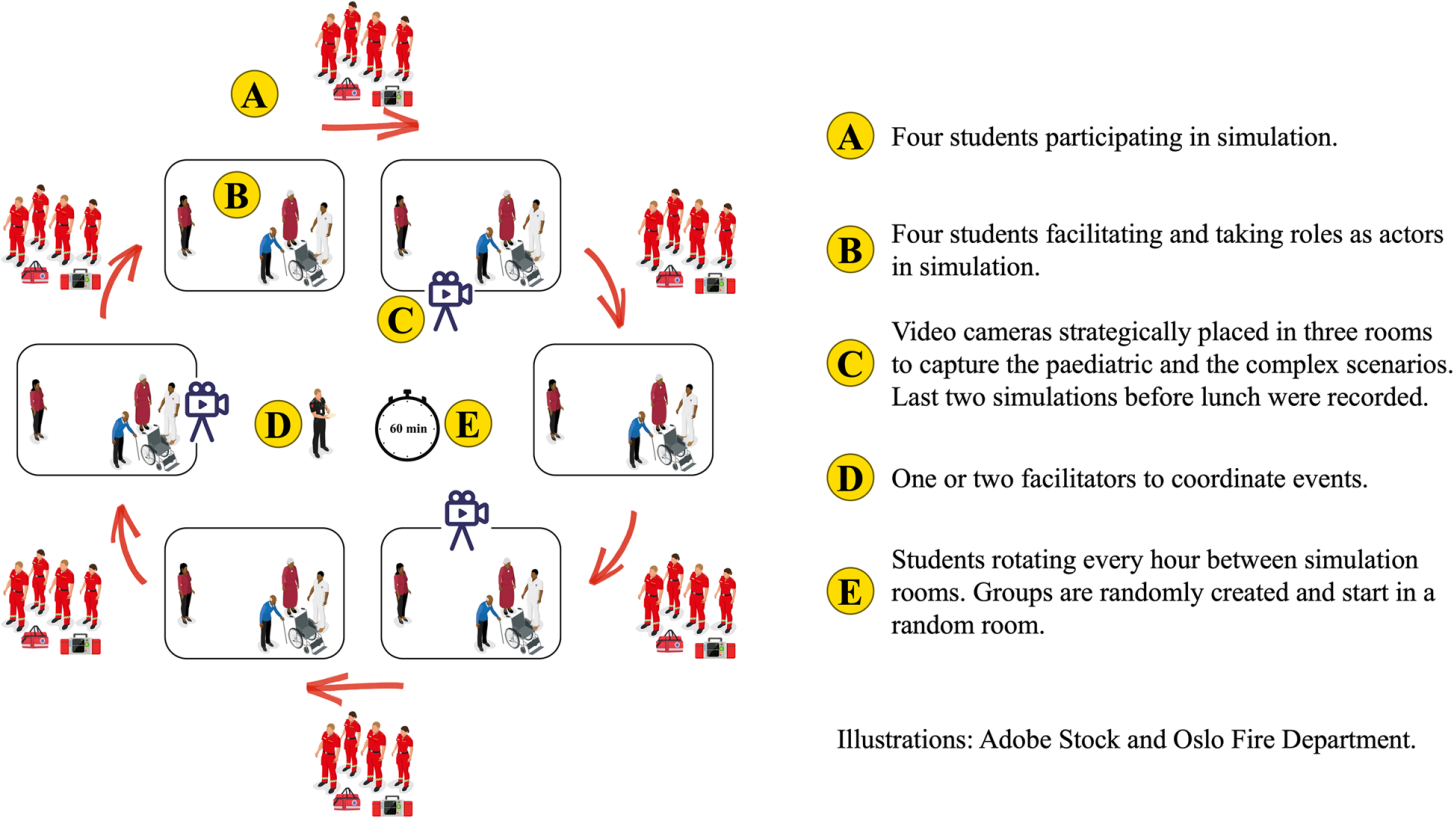
Organisation of student-led simulation days

For student-led simulation, the cohort was divided into 12 groups of about four students. Six groups delivered simulation on the first day, whilst the other half participated in their fellow students’ scenarios. On the second day, the roles were reversed. Student groups would rotate every hour between simulation rooms and get to experience all six scenarios. See Fig. 2 for organisation of student-led simulation.

In preparation, the groups wrote the scenario scripts themselves, and for this, they were assigned a unique topic from the syllabus 3 weeks prior. Defining learning objectives and content within the assigned topic was at the students’ discretion. A scenario script template was provided to the students which some groups used, others not. The students’ scenarios were not reviewed by the faculty. Four different student scenario topics were selected to match those delivered by facilitators. Two of these scenarios could be categorised as paediatric scenarios (5 and 6 in Table 1), and two could be categorised as complex assessments’ scenarios (7 and 8 in Table 1). Twelve debriefings from six different groups were filmed during the 2 days of student-led simulation.

### Data collection and preparation

Video cameras were placed with a good view of the debriefing area clearly showing all debriefing participants. This was chosen over sound-only recording, as it was believed the video would aid in distinguishing speakers during transcription. Those facilitating the scenario, either paramedic facilitator or students, were responsible for starting and stopping the recordings. Transcription was done by an external agency, but the material was also reviewed by the authors.

### Analysis

For the analysis, an adapted version of Fleck’s framework [53] for assessing the level of reflection in statements was used (Table 2). This framework was originally developed for teacher students to reflect upon pictures from their classroom performance and has later been modified for simulation debriefing purposes [54]. In the transcription, each participant’s turn in a dialogue was considered a unit of analysis. Each turn received one rating, and when multiple reflection levels were evident within a turn, only the highest was selected. See Table 3 for examples.

**Table 2:** Reflective framework with adaptions as used here [53, 54]

Definition		Features
R0	Description “A description or statement about events without further elaboration or explanation.”	- Non-reflective - Descriptive - Clarifying - No reasons or justifications given - Short utterances such as “yes, it was”^a^ - Evaluation without explanation^b^
R1	Descriptive reflection “Description including justification or reasons for action, but in a reportive or descriptive way. No alternate explanations explored, limited analysis and no change of perspective.”	- Descriptive with explanation - Evaluation with explanation^b^ - Reasons and justifications for actions, choices, or interpretations ^b^ - Explanations or ideas that are already possessed - Explaining or referencing guidelines and practices known beforehand by the participant^a^ - Suggestion for change - Probing answer ^b^
R2	Dialogic reflection “A different level of thinking about. Looking for relationships between pieces of experience, evidence of cycles of interpreting and questioning, consideration of different explanations, hypothesis and other points of view.”	- Questioning assumptions - Referencing to experiences - Relating experience to theoretical concepts - Interpreting and hypothesising - Considering or suggesting different explanations or alternatives ^b^ - Considering implications of observations, interpretations, and suggestions - Generalising from experience - Probing question with explanation ^b^ - Probing answer with explanation ^b^
R3	Transformative reflection “Revisiting an event with the intent to re-organise and do something differently. Asking fundamental questions and challenging personal assumptions leading to a change in practice.”	- Fundamental questioning of assumptions and motivations - Fundamental change of perspective
R4	Critical reflection “Where social and ethical issues are taken into consideration. Generally considering the (much wider) picture.”	- Ethical - Political - Relating to society, culture and the world as a whole

^a^Additions suggested by Kihlgren et al.

^b^Addition or adjustment used in this study

**Table 3:** Example of coding

Turn	Speaker	Turn content	Identified features	Rating
36	Student 2	We should have auscultated when he became dyspnoeic	- Suggestion for change	R1: Reflective description
37	Student 1	Yes! We should have jumped straight to assessment, and revealed that…	- Suggestion for change	R1: Reflective description
38	Student 2	But we attached the pulse oximeter and saturations did not fall.	- Descriptive - Clarifying	R0: Description
39	Student 3	Saturations didn´t change.	- Descriptive - Clarifying	R0: Description
40	Facilitator	There is nothing wrong in stopping and re-assessing	–	Not rated
41	Student 2	But we did not have any significant findings either. That is why we stepped back and checked saturations, noted he was breathing heavier, and we adjust oxygen flow.	- Explains or justifies actions	R1: Reflective description

Debriefing 21. Translation to English provided by authors

**Fig. 3:**
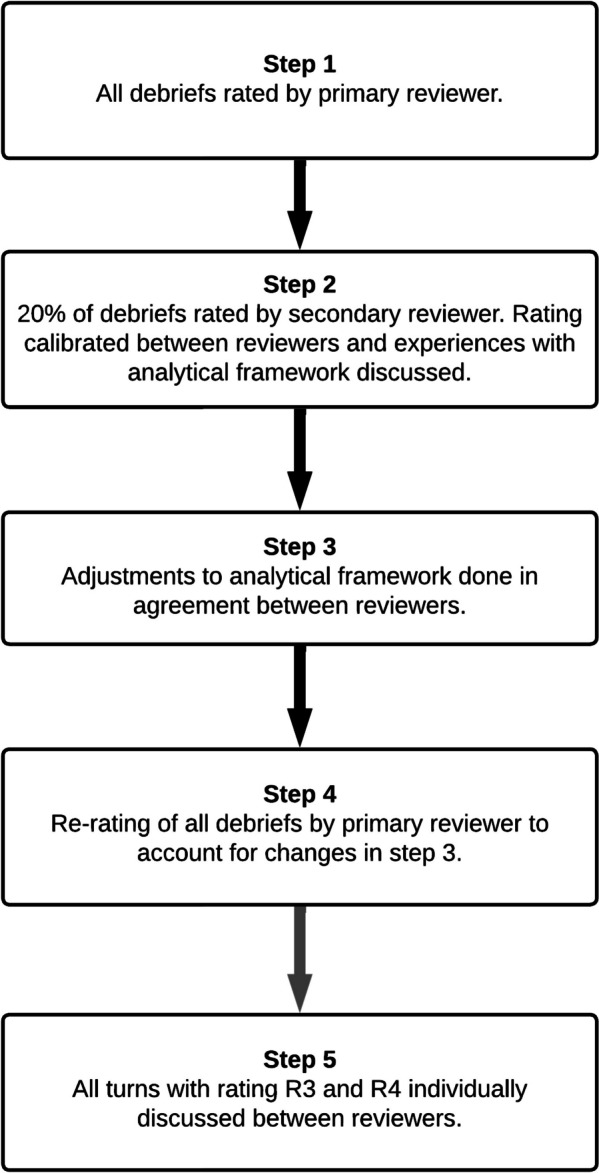
Stepwise approach to data analysis

Analysis was done in a five-step process, as illustrated in Fig. 3. In step 1, all transcripts were rated by the primary reviewer (C.R.C.). The rating was done by finding reflective features within each conversation turn. A reflective feature is one of multiple attributes associated with a level of reflection. When finding such a feature, the turn was rated with the associated level of reflection. An example of the coding process is presented in Table 3. In step 2, 20% of transcripts were rated by a secondary reviewer (J.V.A.), and discrepancy in assessment was discussed and agreed upon. This acted as a calibration of the primary reviewer. Additional features were discovered in the process, and other features needed modification to precisely capture the variations of reflection. Step 3 consisted of additional modifications to the framework, as indicated by ** in Table 2. In step 4, all transcripts were re-rated by the primary reviewer to account for the changes made to the framework. Lastly in step 5, all turns receiving rating R3 and R4 were individually discussed between both reviewers to prevent false high ratings.

Occasionally participants would ask questions of which they truly did not know the answer. For example, a question of factual nature like the correct treatment for a specific condition. These were designated true questions, to not confuse with statements which had the characteristics of a probing question with explanation (a feature of the R2-level of reflection). Probing questions are concealed statements or claims. The reflective framework was made for utterances, not questions. True questions were therefore omitted from the analysis.

Significance testing was done using chi-square test of independence with the Social Science Statistics Calculator [55]. This is an appropriate test for non-parametric data in a 2 × 2 table when variables are categorical, groups are seen as independent single entities, cells are mutually exclusive, and the expected frequency is not less than five [56]. The data meets these assumptions. To allow for significance testing of reflection levels between groups in a 2 × 2 table, the reflection levels were classified as either low or high. R0–R1 were classified as low level, and R2–R4 as high level of reflection. This was based on the findings of Kihlgren et al. who found only 10% of their debriefing contributions to be at R2-level, and none at R3 or R4 [54].

Lauritzen et al. have been concerned about whether certain scenario types were more prone to evoke higher reflection levels than others [57]. The scenarios in this study could be categorised as either critically sick children (paediatric scenarios), or complex assessment scenarios relating to situations with vulnerable and/or multimorbid patients with compound issues of a medical, legal, ethical, social, and/or practical nature (complex scenarios). This categorisation allowed for a separate analysis to test Lauritzen’s ideas.

### Ethical considerations

Written informed consent was obtained from all students and facilitators. As the investigators are lecturers at the same department, which might imply an asymmetrical relationship towards the participants, particular care was taken to create a positive atmosphere so it would be easier for participants to decline participation. The study has been approved by the Norwegian Centre for Research Data (NSD) no. 425765 and the local data protection officer at OsloMet. According to Norwegian legislation, the study is not eligible for review by the regional ethics committee as it is a non-clinical study and contains neither patient nor biomedical data [58, 59]. The study is in accordance with the reporting template for health care simulation research which are extensions to the CONSORT and STROBE statements [60]. The complete report is available in the Appendix 1.

## Results

A total of 22 debriefings were analysed. Of these, 10 were led by a facilitator and 12 were led by a student. The median length of debriefings led by facilitators was 18.0 min (ranging from 10.5 to 22.3 min) and for debriefings led by students 14.9 min (ranging from 8.1 to 26 min). An overview of the debriefings is presented in Table 4. The time allocated for simulation including the debriefing was for the most part 45 min. However, about half of the facilitator-led simulations were allocated 60 min. Consequently, it is difficult to compare the length of debriefings because available time differed between simulations.

**Table 4:** Overview of debriefings

	Facilitator-led	Student-led
Debriefings subject for analysis	10	12
Median length of debriefing	18.0 min	14.9 min
Range length of debriefing	10.5 to 22.3 min	8.1 to 26.0 min

A secondary reviewer rated 20% of the content in terms of reflection levels, and inter-rater reliability was calculated to be 0.67 which is considered fair to good agreement [61].

All contributions in the debriefings made by the students were rated for their level of reflection. Neither contributions by facilitators nor student facilitators were rated. A total of 1384 turns were rated, of these 682 (49%) were in facilitator-led debriefings and 702 (51%) in student-led debriefings. Results are presented in Table 5.

**Table 5:** Distribution of reflection in facilitator-led and student-led debriefings

	Facilitator-led		Student-led	
	%	n	%	n
R0 description	32.7	223	33.8	237
R1 reflective description	44.0	300	44.3	311
R2 dialogical reflection	14.7	100	17.1	120
R3 transformative reflection	0.1	1	1.3	9
R4 critical reflection	0.1	1	0.1	1
Questions	8.4	57	3.4	24
Total	100.0	682	100.0	702

To test the hypothesis that student-led simulation achieved equivalent reflection levels in debriefings as facilitator-led simulation, results were grouped in a 2 × 2 table (Table 6). The Chi-square test showed that the difference in reflection level between facilitator-led and student-led simulation was not significant with a *p* value of > 0.05 (*χ*2 (*df* = 1, *N* = 1303) = 1.81, *p* = 0.178).

**Table 6:** Distribution of reflection grouped in a 2 × 2 table for facilitator-led and student-led debriefing

Level of reflection	Facilitator-led		Student-led	
	%	n	%	n
R0–R1	83.7	523	80.8	548
R2–R4	16.3	102	19.2	130

When organising scenarios according to type, we got 11 paediatric scenarios with a total of 509 (42.7%) turns, and 11 complex scenarios with a total of 793 (57.3%) turns. There were facilitator-led and student-led simulations in both groups. Results are presented in Table 7.

**Table 7:** Distribution of reflection in debriefings in paediatric and complex scenarios

	Paediatric scenarios		Complex scenarios	
	%	*n*	%	*n*
R0 description	35.4	209	31.6	251
R1 reflective description	45.3	268	43.3	343
R2 dialogical reflection	13.4	79	17.8	141
R3 transformative reflection	0.5	3	0.9	7
R4 critical reflection	0.0	0	0.3	2
Questions	5.4	32	6.2	49
Total	100.0	591	100.0	793

To test the hypothesis that students would achieve equivalent levels of reflection regardless of the type of scenario, the scenarios categorised as paediatric and complex were grouped in a 2 × 2 table (Table 8). The chi-square test showed significant differences in reflection levels between paediatric and complex scenarios with a *p* value of < 0.05 (*χ*2 (*df* = 1, *N* = 1303) = 6.58, *p* = 0.010).

**Table 8:** Distribution of reflection grouped in a 2 × 2 table for paediatric and complex scenarios

Level of reflection	Paediatric scenarios		Complex scenarios	
	%	*n*	%	*n*
R0–R1	85.3	477	79.8	594
R2–R4	14.7	82	20.2	150

As a debriefing is a conversation between the facilitator and the simulation participants (the students), their turns in the conversation were counted. During facilitator-led debriefings, the students had on average 62.7% (range 50–77.6%) of the turns to speak. For debriefings led by students, the participating students had 60.6% (range 53.0–76.6%) of the turns to speak. No significant differences were found between the amount of turns to speak when comparing facilitator-led with student-led debriefing using a chi-square test with a *p* value of > 0.05 (*χ*2 (*df* = 1, *N* = 2246) = 1.12, *p* = 0.290). Results are presented in Table 9.

**Table 9:** Distribution of turns to speak based on the number of turns in a debriefing

	Facilitator-led		Student-led	
	%	*n*	%	*n*
Facilitator turns	37.3	405	39.4	457
Student turns	62.7	682	60.6	702

The relationship between the length of turns and reflection levels was assessed (Table 10). With the exception of R4 critical reflection, there seemed to be a pattern where increasing lengths of turns are related to a higher level of reflection.

**Table 10:** Length of turns

	Turns (*n*)	Median number of words
R0 description	460	6
R1 reflective description	611	20
R2 dialogical reflection	220	43
R3 transformative reflection	10	66
R4 critical reflection	2	58
Questions	57	6
Facilitators turn	862	12

## Discussion

This study found no statistically significant differences in reflection levels between facilitator-led and student-led debriefing. On the other hand, differences were found when comparing paediatric to complex scenarios. Complex scenarios had significantly higher reflection levels in the debriefing than paediatric scenarios. The degree of student participation in the debriefing were found to be comparable in facilitator-led and student-led groups.

### Student-led simulation

A possible explanation for the comparable reflection levels between groups is that paramedic students have developed their ability to arrange and debrief simulations through gradually increased participation in simulation activities, a learning process described by Lave and Wenger [14, 62]. Therefore student-led simulation is probably appropriate for simulation-experienced students. This assumption is echoed by an explorative qualitative study on student-led simulation by final-year nursing students. The study identified three success criteria: That students were familiar with simulation, had sufficient content knowledge beforehand, and were in an emotionally safe learning environment [25]. Other studies have demonstrated that anxiety with PAL is prevalent amongst undergraduate students, whilst postgraduate students embrace it [12]. Thus, student seniority might affect anxiety related to PAL. It seems like sufficient simulation experience, sufficient content knowledge, a safe learning environment, and student seniority may be key elements for student-led simulation.

It is unclear what triggers reflection in student-led debriefing. Previous studies have mixed results between debriefers questioning and the reflection levels they elicit in responses [37, 38]. This study was not designed to categorise debriefers’ questions and matching these with reflections in responses, understand if or how debriefing content is related to expected learning outcomes, or provide insight into peer dynamics. Our study helps to establish that student-led debriefings can trigger deep-going reflections, but shed little light on how they actually do that—what kind of interactions are used to which effect. Our study establishes the setting as one in which further studies can explore such interactions in more detail without risking low-quality teaching. A study on eight-grade school children has previously demonstrated a higher level of reasoning and better explanations when discussions were guided by teachers compared to group discussions without teacher influence [50]. Although not generalisable to university students, it indicates that discourse patterns might be of a different nature in different contexts. A possibility is that students who facilitate the debriefing better understand fellow students’ perspectives and challenges, and therefore manage to focus the discussion on the pertinent parts and in this way engage in deep discussions. Taking into consideration that content knowledge possibly is a pre-requisite for student-led activities, it might be that students just stick to talking about things they already are knowledgeable about, while disregarding things they do not know much about [20, 21]. As debriefing is a complex social practice, also debriefers’ actions are an integral part of the debriefing—for example their non-verbal interaction with the students. Our study, again, lays the ground for such research, as it established student-led debriefings as a learning setting that is valuable for students and therefore can be used to explore interaction- and discourse patterns in student-led debriefing without providing low quality teaching.

### Impact of scenario design on reflection levels

Kihlgren et al. were the first to use Fleck’s reflective framework to analyse debriefings following simulation [54]. Like our findings, they found overall little reflection at higher levels. Direct comparison with our material is not possible as they only analysed selected parts of the debriefing, whilst our study analysed the debriefing in its entirety. In addition, as shown in Table 2, the reflective framework has undergone additional changes since its use. They question whether there are features in the scenario design itself that trigger higher reflection levels [54]. Their scenarios involved emergency medical situations (anaphylaxis and septic shock), and they questioned if those scenarios were of an instrumental character where learning goals were associated with R1- and R2-reflection levels [54]. As mentioned previously, also Lauritzen has wondered if some types of scenarios are more likely than others to elicit higher reflection levels [57]. It is uncertain what explains the overall low reflection levels. It might be related to scenario design not triggering reflection, that the overall topic is of a too instrumental character to evoke reflection, that the difficulty level is not high enough, or a combination of multiple factors.

Our study consists of scenarios that could thematically be split in two. On the one hand, paediatric emergency medical cases (paediatric scenarios), with situations like breathing problems, sepsis, or anaphylaxis. On the other, cases consist of situations in vulnerable and/or multimorbid patients with compound issues of a medical, legal, ethical, social, and/or practical nature (complex scenarios). We believed that the paediatric scenarios could be of similar instrumental nature, with clearer advice found in medical literature, as those in Kihlgren et al.’s study. In contrast, we believed that the complex scenarios could possibly carry features in the swampy zones of professional practice [35], and therefore possibly trigger more higher reflection levels. When assessing for this, statistically significant differences were discovered. Complex scenarios achieved more higher-level reflections and proportionally less lower-level reflections, than the paediatric scenarios. The research seems to support Kihlgren et al.’s and Lauritzen et al.’s thoughts that scenario features may play a role in eliciting higher reflection levels [54, 57]. Further research addressing the relationship between dilemmas within scenarios and reflection levels in debriefing would be useful to clarify this.

### Student participation in debriefing

A previous study has reported higher self-reported short-term learning outcomes in debriefings in relation to increased interaction between participants, and lower self-reported learning outcomes when interaction was mostly between debriefer and individual participants [44]. If the degree of participation is associated with increased perceived learning, then what promotes or discourages participation is worthwhile to investigate. We assumed the presence of an asymmetrical relationship between professional facilitators and students [12, 63, 64]. Furthermore, we postulated this asymmetry could lead the facilitator to dominate the talking time in the debriefing, thus reducing the left-over time for students to voice their thoughts [65]. The power-imbalance could potentially also affect students’ willingness to contribute to the conversation [64, 66, 67]. It could for example be that a greater proportion of what is said lies with the facilitator and with very verbal students, while less outspoken students are more hesitant to speak and therefore contribute less to the debriefing. By removing the dominant presence of a professional facilitator, students might feel less restricted and therefore contribute more to the debriefing. We believed this would be measurable by counting the students’ contribution to debriefings and doing a comparison between facilitator-led and student-led debriefing. If simulation participants speak more often in one of the groups, it may be a sign that they more easily get the floor to speak. However, this investigation failed to find any significant differences in the amount of turns to speak by students who participated in the simulation, regardless of whether the debriefing was led by a facilitator or a student. It should be noted that group composition affects our understanding of the results. In facilitator-led simulations, there was one facilitator and six students. In student-led simulations, there were three facilitating students and three participating students. Hence, in facilitator-led simulations, 62.7% of the turns came from six students. On the other hand, in student-led simulations, 60.6% of the turns came from only three students. Another area of exploration could be whether teacher presence affects various students differently. Maybe outspoken students are vocal regardless of the facilitator presence, and less outspoken students speak more freely in the absence of an authority figure [10]. Mapping speakers in a sociogram could possibly be another approach to investigate this [44, 68, 69]. Unfortunately, this was outside the remits of this research project. Since increased interaction seems to be associated with increased perceived learning [44], this warrants further exploration in student-led simulation.

### Cost considerations

We have worked along two lines of argument on student-led simulation. So far we have discussed the first, on the pedagogical value of student-led simulation. The second line of argument concerns costs. A full economic analysis is beyond the scope of this article; however, we wish in simple terms to shed light on this with our model as an example. We also acknowledge that the calculation of cost-saving can differ between institutions. In our centre, 2 days of student-led simulations resulted in 72 simulations. This requires six working days: One facilitator for 2 days to prepare students for the event, and two facilitators on site for the 2 days of simulation. The same amount of simulations run by facilitators would have required 12 working days: four facilitators over 3 days of simulations. This is a substantial saving in terms of work hours. Additional savings might be materialised when the students later want to become simulation faculty themselves. They already would have simulation facilitator experience and could thus be trained more quickly than if they had no experience. Involving students in the operation of a simulation centre has many benefits that our group has discussed in a previous paper [70], including for example finding future faculty, networking, research, operators, and more.

### Limitations

This study has been limited to investigating levels of reflection in facilitator-led and student-led simulations. However, as mentioned in the introduction, debriefing consists of other aspects like the debriefing process, quality and appropriateness of content discussed, and psychological safety amongst participants to name but a few examples. Applying student-led debriefings has an impact on and will be impacted by the whole simulation setting [46]. It needs to be prepared on an organisational (curricular), team-based (the simulation center), and individual (facilitators and students) level. It is reasonable to assume learning also has taken place for students organising simulation, as teaching others is a great way of learning [25, 49, 71, 72]. Having to construct their own scenarios and seen multiple solutions with debriefings to the same scenario, they have likely developed a greater understanding of their topic. Only students undergoing simulation have been the focus of this investigation, and future studies should address the learning of the students providing simulation. There seems to be a lot of creativity, joy, and learning that comes to light through scenarios created by students. There might also be downsides like performance pressure for students facilitating and for students participating in simulation. All of this has not been captured in this work. An avenue for further exploration is the potential association with self-organised simulation and gained competency in simulation facilitation, and importantly if this could lead to increased simulation activity throughout a career.

A limitation of this study is the lack of randomisation and use of a control group, which makes it more prone to bias and confounders, and no causal relationship can be established [73]. Another limitation is that although the facilitator-led and student-led simulations had the same topics and characteristics, the actual scenarios were different. The paediatric scenarios were all about medical emergencies but in facilitator-led simulations, they were about septic shock and hypoglycaemia, whilst in the student-led they were about the difficulty in breathing and reduced consciousness. The same applied to the complex scenarios, which were all related to vulnerable and/or multimorbid patients, but their actual conditions and complicated needs differed. Further, it was not possible to blind reviewers to whether the debrief was led by a facilitator or a student, as the transcription carried clear evidence of what kind of simulation had taken place. Data collection was done overtly, and participants themselves initiated and stopped recordings. This could have contributed to a Hawthorne effect [74] influencing behaviours like willingness to share thoughts about own mistakes or perceived own inadequacies. This should not negate the ability to compare groups, as this would presumably affect both groups equally. These results can only be applied to simulation-experienced paramedic students at OsloMet. Generalisation to other contexts or to simulation-naïve students should be done with caution.

The ability of the framework to capture actual reflection, and thus the validity of the results, can be questioned. According to Fleck, any tool measuring reflection measures only what is overt, and not what is in the person’s mind [53]. For example, all kinds of non-verbal reflections—diffuse and yet clear feelings, are not captured.

An instrument like the one in this study should produce comparable results, regardless of who uses it. Inter-rater reliability was calculated to be 0.67 for 20% of the dataset. This is accepted to be fair to good agreement between reviewers [61]. Increased reliability has been attempted to achieve by calibrating the primary reviewer with feedback from the secondary reviewer. Two independent raters for the whole dataset were outside the resource possibility of this study. Furthermore, to prevent false high ratings, all R3- and R4-level ratings received an additional joint review by both reviewers.

## Conclusions

This study has shown that simulation-experienced paramedic students can lead debriefings with comparable reflection levels as trained and experienced paramedic facilitators. As the students’ debriefing are the results of their self-arranged simulations, it is reasonable to assume simulation-experienced students also can plan and deliver simulation events on their own. These results are important as they offer an additional approach to simulation in healthcare education. Student-led simulation offers the benefit of PAL. It may also require less teacher resources reducing the costs of running simulations. This could lead to increased simulation frequency, and using available simulation facilities in a more efficient way. Furthermore, this research has found that scenario design might influence reflection levels in the debriefing. More research is needed to explore which features within scenario design trigger higher reflection levels. Lastly, we did not find that facilitator presence impacted the degree of student participation in the debriefing sessions. This study adds to the repository of studies looking into reflection levels in debriefing following simulation events.

### Supplementary Information

**Additional file 1:** **Appendix 1.** Adherence to reporting guidelines for simulation-based research This article complies with the reporting guidelines for health care simulation, which are extensions to the CONSORT and STROBE statements (32).

## Data Availability

The dataset is available from the corresponding author on reasonable request.

## Abbreviations

NSD: Norwegian Centre for Research Data
PAL: Peer-Assisted Learning
OsloMet: Oslo Metropolitan University

## References

[1] Society for Simulation in Healthcare. About Simulation 2022 [Available from: https://www.ssih.org/About-SSH/About-Simulation. Accessed: 01.09.2022

[2] Durham CF, Alden KR. Enhancing patient safety in nursing education through patient simulation. Patient safety and quality: an evidence-based handbook for nurses 2008. 21328731

[3] Wang EE (2011). Simulation and adult learning. Disease-a-month..

[4] Boese T, Cato M, Gonzalez L, Jones A, Kennedy K, Reese C (2013). Standards of best practice: simulation standard V: Facilitator. Clin Simul Nurs..

[5] Cheng A, Morse KJ, Rudolph J, Arab AA, Runnacles J, Eppich W (2016). Learner-centered debriefing for health care simulation education: lessons for faculty development. Simul Healthc..

[6] Maran NJ, Glavin RJ (2003). Low-to high-fidelity simulation–a continuum of medical education?. Med Educ..

[7] Motola I, Devine LA, Chung HS, Sullivan JE, Issenberg SB (2013). Simulation in healthcare education: a best evidence practical guide. AMEE Guide No. 82. Med Teach..

[8] Zendejas B, Wang AT, Brydges R, Hamstra SJ, Cook DA (2013). Cost: the missing outcome in simulation-based medical education research: a systematic review. Surgery..

[9] Tolsgaard MG (2013). Clinical skills training in undergraduate medical education using a student-centered approach. Dan Med J..

[10] Olaussen A, Reddy P, Irvine S, Williams B. Peer-assisted learning: time for nomenclature clarification. Med Educ Online. 2016; 10.3402/meo.v21.30974 PMC4944593 27415590

[11] Tolsgaard MG, Gustafsson A, Rasmussen MB, HØiby P, Müller CG, Ringsted C (2007). Student teachers can be as good as associate professors in teaching clinical skills. Med Teach..

[12] Gazula S, McKenna L, Cooper S, Paliadelis P (2017). A systematic review of reciprocal peer tutoring within tertiary health profession educational programs. Health Professions Educ..

[13] Topping KJ (2005). Trends in peer learning. Educ Psychol..

[14] Topping KJ (1996). The effectiveness of peer tutoring in further and higher education: a typology and review of the literature. High Educ..

[15] Eddy ER, Tannenbaum SI, Mathieu JE (2013). Helping teams to help themselves: comparing two team-led debriefing methods. Pers Psychol..

[16] Roh YS, Kelly M, Ha EH (2016). Comparison of instructor-led versus peer-led debriefing in nursing students. Nurs Health Sci..

[17] Boet S, Bould MD, Bruppacher HR, Desjardins F, Chandra DB, Naik VN (2011). Looking in the mirror: Self-debriefing versus instructor debriefing for simulated crises. Crit Care Med..

[18] Boet S, Bould MD, Sharma B, Revees S, Naik VN, Triby E (2013). Within-team debriefing versus instructor-led debriefing for simulation-based education: a randomized controlled trial. Ann Surg..

[19] Kim SS, De Gagne JC (2018). Instructor-led vs. peer-led debriefing in preoperative care simulation using standardized patients. Nurse Educ Today..

[20] Stasser G, Titus W (1985). Pooling of unshared information in group decision making: biased information sampling during discussion. J Pers Soc Psychol..

[21] Wittenbaum GM, Stasser G (1996). Management of information in small groups.

[22] Leigh GT, Miller LB, Ardoin KB (2017). A nurse educator's guide to student-led debriefing. Teach Learn Nurs..

[23] Levett-Jones T, Lapkin S (2014). A systematic review of the effectiveness of simulation debriefing in health professional education. Nurse Educ Today..

[24] Babla K, Lipton J, Williams S, Chopra P, Thenabadu S (2020). Simprovisation: a model for student-led simulation. Clin Teach..

[25] Svellingen A, Røssland A, Røykenes K (2021). Students as facilitators: experiences of reciprocal peer tutoring in simulation-based learning. Clin Simul Nurs..

[26] Nunnink L, Thompson A (2018). Peer-assisted learning in scenario-based simulation. Med Educ.

[27] Jauregui J, Bright S, Strote J, Shandro J (2018). A novel approach to medical student peer-assisted learning through case-based simulations. West J Emerg Med..

[28] Curtis E, Ryan C, Roy S, Simes T, Lapkin S, O'Neill B (2016). Incorporating peer-to-peer facilitation with a mid-level fidelity student led simulation experience for undergraduate nurses. Nurse Educ Pract..

[29] Carey JM, Rossler K (2020). The how when why of high fidelity simulation. [Updated 2023 May 1]. In: StatPearls.

[30] Tamkin P, Yarnall J, Kerrin M (2002). Kirkpatrick and beyond: a review of models of training evaluation: Institute for Employment Studies Brighton, England.

[31] Atkins S, Murphy K (1993). Reflection: a review of the literature. J Adv Nurs..

[32] Mamede S, Schmidt HG (2004). The structure of reflective practice in medicine. Med Educ..

[33] Nguyen QD, Fernandez N, Karsenti T, Charlin B (2014). What is reflection? A conceptual analysis of major definitions and a proposal of a five-component model. Med Educ..

[34] Amulya J (2004). What is reflective practice. Center for Reflective Community Practice.

[35] Schön DA (1987). Educating the reflective practitioner.

[36] Kainth R, Reedy G. Transforming Professional Identity in Simulation Debriefing: A Systematic Metaethnographic Synthesis of the Simulation Literature. Simul Healthc. 2023;10(1097) 10.1097/SIH.0000000000000734 37335122

[37] Kolbe M, Grande B, Lehmann-Willenbrock N, Seelandt JC (2023). Helping healthcare teams to debrief effectively: associations of debriefers’ actions and participants’ reflections during team debriefings. BMJ Qual Saf..

[38] Husebø S, Dieckmann P, Rystedt H, Søreide E, Friberg F (2013). The Relationship between facilitators’ questions and the level of reflection in postsimulation debriefing. Simulation in Healthcare, 8 (3), 135-142. Clin Simul Nurs..

[39] Dieckmann P, Gaba D, Rall M (2007). Deepening the theoretical foundations of patient simulation as social practice. Simul Healthc..

[40] Dieckmann P, Sharara-Chami R, Ersdal HL (2020). Debriefing practices in simulation-based education. Clinical Education for the Health Professions: Theory and Practice.

[41] Ko E, Choi Y-J (2020). Debriefing model for psychological safety in nursing simulations: a qualitative study. Int J Environ Res Public Health..

[42] Kolbe M, Eppich W, Rudolph J, Meguerdichian M, Catena H, Cripps A (2020). Managing psychological safety in debriefings: a dynamic balancing act. BMJ Simul Technol Enhanced Learn..

[43] Dieckmann P, Molin Friis S, Lippert A, Østergaard D (2009). The art and science of debriefing in simulation: Ideal and practice. Med Teach..

[44] Abegglen S, Greif R, Balmer Y, Znoj HJ, Nabecker S (2022). Debriefing interaction patterns and learning outcomes in simulation: an observational mixed-methods network study. Adv Simul..

[45] Raemer D, Anderson M, Cheng A, Fanning R, Nadkarni V, Savoldelli G (2011). Research regarding debriefing as part of the learning process. Simul Healthc..

[46] Dieckmann P, Birkvad Rasmussen M, Issenberg S, Søreide E, Østergaard D, Ringsted C (2018). Long-term experiences of being a simulation-educator: a multinational interview study. Med Teach..

[47] Collier KG (1980). Peer-group learning in higher education: The development of higher order skills. Stud High Educ..

[48] Adelopo I, Asante J, Dart E, Rufai I (2017). Learning groups: The effects of group diversity on the quality of group reflection. Acc Educ..

[49] Tai J, Molloy E, Haines T, Canny B (2016). Same-level peer-assisted learning in medical clinical placements: a narrative systematic review. Med Educ..

[50] Hogan K, Nastasi BK, Pressley M (1999). Discourse patterns and collaborative scientific reasoning in peer and teacher-guided discussions. Cogn Instr..

[51] Hahn S (2012). Understanding noninferiority trials. Korean J Pediatr..

[52] Steinwachs B (1992). How to facilitate a debriefing. Simul Gaming..

[53] Fleck R (2012). Rating reflection on experience: a case study of teachers’ and tutors’ reflection around images. Interact Comput..

[54] Kihlgren P, Spanager L, Dieckmann P (2015). Investigating novice doctors' reflections in debriefings after simulation scenarios. Med Teach..

[55] Stangroom J. Chi-square test calculator. Social Science Statistics. 2022 [Available from: https://www.socscistatistics.com/tests/chisquare2/default2.aspx. Accessed: 20.10.2022

[56] Pallant J, Pallant J (2020). SPSS survival manual : a step by step guide to data analysis using IBM SPSS. 7th edition. ed.

[57] Lauritzen J (2016). Reflection levels in simulation-based training.

[58] The Health Research Act. ACT 2008-06-20 no. 44: Act on medical and health research (the Health Research Act) In: The Norwegian Ministry of Health and Care Services, editor. 2008.

[59] The Regional Ethics Committe. About Applying to REK 2022 [Available from: https://rekportalen.no/#hjem/s%C3%B8ke_REK. Accessed: 01.10.2022

[60] Cheng A, Kessler D, Mackinnon R, Chang TP, Nadkarni VM, Hunt EA (2016). Reporting guidelines for health care simulation research: extensions to the CONSORT and STROBE statements. Adv Simul..

[61] Fleiss JL, Levin B, Paik MC (2003). Statistical methods for rates and proportions.

[62] Lave J, Wenger E (1991). Situated learning: legitimate peripheral participation.

[63] Tosterud R, Kjølberg K, Kongshaug AV, Haugom JV (2020). Exploration of two different structures for debriefing in simulation: The influence of the structure on the facilitator role. Simul Gaming..

[64] Baker-Rush ML, Pabst A, Aitchison R, Anzur T, Paschal N (2021). Fear in Interprofessional Simulation: The role of psychology and behaviorism in student participation and learning. J Interprofessional Educ Pract..

[65] Ulmer FF, Sharara-Chami R, Lakissian Z, Stocker M, Scott E, Dieckmann P (2018). Cultural prototypes and differences in simulation debriefing. Simul Healthc..

[66] Rana S-C, Francis U, Zavi L, Ella S, Honein-Abou Haidar G, Peter D. Cultural differences in simulation debriefing: A qualitative analysis. Heliyon. 2023;9(4) 10.1016/j.heliyon.2023.e14904 PMC10102195 37064463

[67] Robertson K, Ju M, O’Brien BC, van Schaik SM, Bochatay N. Exploring the role of power during debriefing of interprofessional simulations. J Interprof Care. 2022:1–9. 10.1080/13561820.2022.2029371 35109751

[68] Simmons J. A better route with conversation maps. Association for Supervision and Curriculum Development. 2020; 77, No. 7(10.09.2022).

[69] Krahenbuhl KS (2020). In class discussions, slow and steady wins. Educ Leadersh..

[70] Viggers S, Østergaard D, Dieckmann P (2020). How to include medical students in your healthcare simulation centre workforce. Adv Simul..

[71] Cortese CG (2005). Learning through teaching. Manag Learn..

[72] Nunnink L, Thompson A, Alsaba N, Brazil V (2021). Peer-assisted learning in simulation-based medical education: a mixed-methods exploratory study. BMJ Simul Technol Enhanc Learn..

[73] Hess AS, Abd-Elsayed A (2019). Observational studies: uses and limitations.

[74] Befring E (2002). Forskningsmetode, etikk og statistikk.

