# Supplementary material for: Comparing reflection levels between facilitator-led and student-led debriefing in simulation training for paramedic students
Source: Adv Simul (Lond). 2023 Dec 14;8:30. doi: 10.1186/s41077-023-00273-0 (PMC10722852; doi:10.1186/s41077-023-00273-0)
Supplement: Supplementary file 1 — Additional file 1: Appendix 1. Adherence to reporting guidelines for simulation-based research This article complies with the reporting guidelines for health care simulation, which are extensions to the CONSORT and STROBE statements (32). [file 41077_2023_273_MOESM1_ESM.docx]

## Appendix: Adherence to reporting guidelines for simulation-based research

This article complies with the reporting guidelines for health care simulation, which are extensions to the CONSORT and STROBE statements (32).

| Participation orientation | | |
| --- | --- | --- |
| *Element* | *Facilitator-led* | *Student-led* |
| Orientation to the simulator | Students have had a minimum of 35 days at the simulation and skills lab prior to this research. Students are well acquainted with simulation and medical equipment, and modus operandi of simulation play. | |
| Orientation to the environment |  |  |
|  |  |  |
| Simulator type | | |
| *Element* | *Facilitator-led* | *Student-led* |
| Simulator make and model | Mostly students acting as patients themselves, for some scenarios simple BLS manikins from Laerdal Medical have been used (Resusci Junior QCPR^®^ with advanced airway and ALS Baby^®^). | |
| Simulator functionality | Isimulate REALITi^®^ monitor-/defibrillator simulator providing spO_2_, etCO_2_, BP and ECG. | |
|  |  |  |
| Simulation environment | | |
| *Element* | *Facilitator-led* | *Student-led* |
| Location | In dedicated simulation facilities at a university simulation centre. | |
| Equipment | Paramedic level portable emergency equipment: Response bag, oxygen, drugs, trolley-bed, PPE, monitor/defibrillator (Isimulate^®^) and telephone. | |
| External stimuli | Music or noise in the background on select cases, where relevant for scenario. | |
|  |  |  |
| Simulation event/scenario | | |
| *Element* | *Facilitator-led* | *Student-led* |
| Event description | Four different scenarios:   1. Child with septic shock 2. Child with hypoglycaemia 3. Nursing home resident with complex needs 4. Frail geriatric patient refusing help after fall | Students develop own scenarios based on assigned topics:   1. Geriatric patient with complex needs 2. Vulnerable patient group 3. Difficulty of breathing in children 4. Reduced level of consciousness in children |
| Learning objectives | Learning objectives focusing on assessment, decision-making and treatment of the medical condition, including ethical, legal, and practical aspects. Learning objectives are derived from learning outcomes from the module description. Learning objectives are defined first, and scenario is scripted to target the objectives. | Learning objectives defined by students themselves, but within the given topic. |
| Group vs. individual practice | Groups of 4-5 students | Groups of 3-4 students |
| Use of adjuncts | Props vary, pending scenario. Moulage make-up for actors, uniforms / clothing appropriate for role, background noise appropriate for scenario, and various props relevant for scenario content (patient medicines, medical documents, alcohol bottles, old food, props for immersive purposes, and there alike). | |
| Facilitator/operator characteristics | Clinically active paramedics, with a three-day facilitator course, and from one to four years’ experience as part-time facilitator at the university. | Third- and final year paramedic students having undergone at least 35 days with simulation and skills training at the university. |
| Pilot testing | Scenarios run 10 times a year and refined over the previous six years. | None. |
| Actors/confederates/standardised/simulated patients | Mostly students acting as patients, for some scenarios simple BLS manikins. Facilitators might act as consultants or counterparts for select scenarios. | Mostly students acting as patients, and students acting as next-of-kin, member of public or other health care professionals. For some scenarios simple BLS manikins. |
|  |  |  |
| Instructional design | | |
| *Element* | *Facilitator-led* | *Student-led* |
| Duration | Total time set for briefing, scenario play and debriefing either 45 or 60 minutes, depending on scenario. | 45 minutes in total set for briefing, scenario play and debriefing. |
| Timing | Data-collection was video-recording of the debriefing, which was performed immediately following the scenario play. | |
| Frequency / repetitions | Students played scenarios only once. | Students played scenarios only once. Students organising the scenario, arranged it six times in a row. |
| Clinical variations | No variation within each scenario, but all scenarios differed amongst each other. | No variation within each scenario, but all scenarios differed amongst each other. |
| Standards / assessments | No assessment. | No assessment. |
| Adaptability of intervention | At facilitator´s discretion, but in practice little need as student group is relative homogenous. | At organising students’ discretion, but in practice little need as student group is relative homogenous. |
| Range of difficulty | Only minor opportunity for facilitator to vary difficulty within the scenario script. Homogenous student groups have relative consistency in performance, and scenarios are calibrated to meet this. | At organising students design and discretion. This area has not been assessed on a scenario-by-scenario basis. |
| Non-simulation interventions and adjuncts | Simulation is part of a 10 ECTS module in emergency medicine running over 10 weeks. Module consists of lectures, workshops, skill-stations, written assignments, individual study time and 8 simulation days per student. Reading list is of approximately 750 pages. On the actual simulation days, only simulations are delivered without any additional interventions. | |
| Integration | Scenarios are scripted on learning objectives, which are defined by the modules learning outcomes. | Topics are allocated to students based on modules learning outcomes and curriculum content. |
|  |  |  |
| Feedback and/or debriefing | | |
| *Element* | *Facilitator-led* | *Student-led* |
| Source | Feedback given from facilitator and peer-students (both participating and observing). | At organising students’ discretion, but in practice feedback is given from facilitating (peer-) students, and peer-students (both participating and observing). No faculty present during debriefing. |
| Duration | 45 or 60 minutes is allocated for simulation event in total (briefing, simulation, and debriefing). Debriefing time at facilitators discretion. Median length 18.0 minutes (range 10.5-22.3 min). | 45 minutes is allocated for simulation event in total (briefing, simulation, and debriefing). Debriefing time at facilitating students’ discretion. Median length 14.9 minutes (range 8.1-26 min). |
| Facilitator presence | One facilitator present for the whole duration. | Three to four students collaboratively organising simulation event. Including facilitating, acting as patients, and filling other roles. Shared facilitator responsibility. Debrief could be led by a student alone, by two, or all three jointly, at students’ discretion. |
| Facilitator characteristics | Clinically active paramedics, employed on an hourly basis as simulation facilitators. Activity ranges between 100-600 hours of simulation- and skill-related work per year. All have a three-day course on how to facilitate simulation and debriefing. | Third- and final year paramedic students without formal training on how to facilitate simulation. Have undergone at least 35 days with simulation and skills training. |
| Content | Focus for learning is primarily medical topics, including pathophysiology, assessment, decision-making, and management. This might also incorporate legal, ethical, or practical aspects. No explicit focus on human factors or environment. | |
| Structure/method | Facilitators trained in using the Steinwachs (24) model for debriefing. Scenario scripts also have a debriefing guide written according to the same model. | No instruction given. Approach to debriefing at students’ discretion. |
| Timing | Debriefing held immediately after simulation, without a break. | |
| Video | Video not used for debriefing purposes, only to collect study data. | |
| Scripting | All scenarios are scripted according to a standardised template. Written language is Norwegian. Scripts available upon request to authors. | All scenarios scripted, but not necessarily according to provided standardised template. Script format at students’ discretion. Scripts unavailable, as they were not collected. |
